# Dyslipidemia in Patients with Xanthelasma Palpebrarum Visiting the Department of Dermatology of a Tertiary Care Centre: A Descriptive Cross-sectional Study

**DOI:** 10.31729/jnma.7485

**Published:** 2022-06-30

**Authors:** Anjan Rai, Sunita Karki, Shree Prasad Sah, Laxmi Narayan Kamat, Manish Pradhan

**Affiliations:** 1 Department of Dermatology, Nobel Medical College and Teaching Hospital, Kanchanbari, Biratnagar, Nepal

**Keywords:** *dyslipidemia*, *gender*, *lipid*

## Abstract

**Introduction:**

Xanthelasma palpebrarum refers to lipid deposition that occurs on eyelids and inner canthi. It is an important cutaneous manifestation of hyperlipidemia, atherosclerosis and coronary artery disease. Few studies have been done in Nepal regarding lipid abnormality in xanthelasma patients. The aim of this study was to find out the prevalence of dyslipidemia among patients with xanthelasma palpebrarum visiting the Department of Dermatology of a tertiary care centre.

**Methods:**

This is a descriptive cross-sectional study conducted among 80 patients from January, 2021 to February, 2022 in the Department of Dermatology of a tertiary care centre. Ethical approval was taken from the Institutional Review Committee (Reference number: 60512021). Convenience sampling was used. Lipid profile analysis was done among patients with clinical diagnosis of xanthelasma palpabrarum. Data was collected using Microsoft Excel for Mac version 16.16.27 and analyzed using the Statistical Package for the Social Sciences version 22.0. Point estimate at 90% Confidence Interval was calculated along with frequency and proportion for binary data.

**Results:**

Out of 80 patients with xanthelasma palpebrarum, the prevalence of dyslipidemia was 64 (80.00%) (74-86 at 90% Confidence Interval). Among them, 29 (45.31%) were males and 35 (54.69%) were females.

**Conclusions:**

In our study, the prevalence of dyslipidemia among patients with xanthelasma palpebrarum was found to be higher than in similar studies conducted in similar settings.

## INTRODUCTION

Xanthoma are a common manifestation of lipid abnormality, the most common of which is Xanthelasma Palpebrarum (XP). It manifests as symmetrical, soft, yellowish, velvety papules and plaques on the lower and upper eyelids.^1^ The cause of XP is unclear, but have hypothesized hormonal, local and macrophage factors in its pathogenesis.^2^ xanthelasma are composed of xanthoma cells which are foamy histiocytes laden with intracellular fat deposits primarily within the upper reticular dermis.^3^ The increased plasma lipid peroxidation might lead to accumulation of cholesterol in macrophages and formation of foam cells via this mechanism.^4^

Most of the xanthelasma occur in normolipemic persons who may have low- or high-density lipoprotein and cholesterol levels or other lipoprotein abnormalities.^5^ XP has been shown to have association with plasma lipid abnormality, its presence might suggest an underlying disorder of lipid metabolism.^6,7^

The study aimed to find out the prevalence of dyslipidemia among patients with xanthelasma palpebrarum visiting the Department of Dermatology of a tertiary care centre.

## METHODS

A descriptive cross-sectional study was conducted in the Outpatient Department (OPD) of Department of Dermatology, Nobel Medical College Teaching Hospital from January, 2021 to February, 2022. This study was started after acquiring ethical approval from the Institutional Review Committee of Nobel Medical College (Reference number: 60512021). All the patients presenting to dermatology OPD within the time frame with clinical diagnosis of xanthelasma palpebrarum were included in the study. Patients having diseases known to be associated with dyslipidemia, patients taking medication known to cause dyslipidemia, pregnant and lactating mothers were excluded from the study. Convenience sampling was done. The sample size was calculated using the formula:


$$\begin{array}{ll} \text{n} & ={\text{Z}}^{2}\times \frac{\text{p}\times \text{q}}{{\text{e}}^{\text{2}}}\\ & =1.645^{2}\times \frac{0.091\times 0.909}{0.06^{2}}\\ & =63 \end{array}$$

Where,

n = minimum required sample size
Z = 1.645 at 90% Confidence Interval (CI)
p = prevalence of dyslipidemia among patients with xanthelasma palpebrarum, 9.1%^4^
q = 1-p
e = margin of error, 6%

The calculated sample size was 63. Taking a 10% non-response rate, the sample size was 70. However, we included 80 patients with a clinical diagnosis of xanthelasma palpebrarum in the study. Relevant history taking and examination was done and after selecting the patients, they were sent for analysis of lipid profile: Total Cholesterol (TC), Low Density Lipoprotein (LDL), High Density Lipoprotein (HDL) and Triglycerides (TG)) in the laboratory of Nobel Medical College. Determination of HDL, LDL, TG and TC was done by an auto analyzer (Lab Systems). The presence of dyslipidemia was defined as a TG of more than 150 mg/dl, TC of more than 200 mg/dl, or LDL of more than 130 mg/dl.^8^

Data collected was entered in Microsoft Excel for Mac version 16.16.27 and analyzed using Statistical Package for the Social Sciences version 22.0. Point estimate at 90% CI was calculated along with frequency and proportion for binary data.

## RESULTS

Out of 80 patients with xanthelasma palpebrarum, the prevalence of dyslipidemia was 64 (80.00%) (74-86 at 90% Confidence Interval). Most of the patients with xanthelama palpabrarm with dyslipidemia had bilateral upper eyelid involvement (Table 1).

**Table 1:** Site of involvement of xanthelasma palpebrarum (n = 64).

Anatomical site	Total n (%)
Bilateral upper eyelid	30 (46.88)
Left upper eyelid	15 (23.44)
Right upper eyelid	18 (28.13)
Bilateral upper and lower lid	1 (1.56)

Among patients with xanthelasma palpebrarum dyslipidemia was more seen in females 35 (54.69%) than males 29 (45.31 %) (Table 2).

**Table 2:** Dyslipidemia in patients with xanthelasma palpebrarum (n = 64).

Sex	n (%)
Male	29 (45.31)
Female	35 (54.69)

Dyslipidemia was present in 17 (26.56%) patients in the age group 46-55 years followed by 14 (21.87%) patients in the age group 56-65 years, 13 (20.31%) patients each in the age group 36-45 years and above 65 years, 5 (7.81%) patients in the age group 26-35 years, 2 (3.12%) patients below 25 years (Table 3). The mean age of patients was 53.28±14.61 years.

**Table 3:** Distribution of patients with dyslipidemia according to age groups (n = 64).

Age group (years)	n (%)
<25	2 (3.12)
26-35	5 (7.81)
36-45	13 (20.31)
46-55	17 (26.56)
56-65	14 (21.87)
>65	13 (20.31)

Dyslipidemia was present in 37 (57.81%) patients with duration of illness 6 months to 1 year followed by 17 (26.56%) patients below 6 months (Table 4).

**Table 4:** Duration of illness among patients with dyslipidemia (n = 64).

Duration of illness	n (%)
<6 months	17 (26.56)
6 months-1 years	37 (57.81)
1-2 years	5 (7.81)
>2 years	5 (7.81)

The mean serum levels of TG, TC, HDL, and LDL values were 170.29±54.07, 200.53±27.09, 42.54±13.94 and 111.31±54.07 respectively.

## DISCUSSION

Xanthelasma has been considered as a marker for dyslipidemia, atherosclerosis, which is the main factor of cardiovascular diseases. Recently the incidence of metabolic diseases tends to increase due to lifestyles, dietary habits and metabolic disorders which also increases the incidence of xanthelasma.^7^ It might predict the possibility of someone acquiring coronary artery disease due to dyslipidemia. Therefore, the occurrence of xanthelasma in an individual should make health workers aware of dyslipidemia and atherosclerosis.^8^

Our study showed female preponderance of (54.69%) as compare to male (45.31%) which was consistent with the findings in another study showing higher percentage of female (80.95%) than male (19.04%).^9^ A study done in Brazil also had a result showing higher percentage of female (85.7%) than males (14.3%).^2^ A study done in China concluded that xanthelasma was more commonly found in female than male.^10^ Theoretically, the predominance of xanthelasma in females is due to hormonal factor (estrogen) in the etiopathogenesis of xanthelasma and the deeper concern to cosmetics in females.^11^

In this study, 46.88% patients had bilateral upper eyelid involvement, 28.13% patients right upper eyelid involvement and 23.44% patients had left upper eyelid involvement. This was similar to another study in which most of the patients had bilateral upper eyelids involvement.^12^ A different study reported that the majority of patients (72.22%) suffered from multiple lesions affecting two or more eyelids.^8^ A study done in India also observed that most of the patients (45.25%) had bilateral upper eyelids involvement.^14^ This finding was comparable to the present study in which involvement of multiple eyelids was found to be 43.8%.

A previous study noted that the highest number of cases 30% were in the age group of 31-40 years.^13^ Similarly, another study also observed that the majority of the patients were in the age group 31-50 years (37.9%).^4^ Published literature had shown that the group with the highest prevalence of xanthelasma was 41-50 years old (38.89%).^8^ This finding was contrary to our study in which the highest number of cases (26.56%) were in the age group 46-55 years.

In a reported study, the mean ages of the patient were 47.4±9.97 years and in most patients (69%) duration of xanthelasma lesions was less than one year,^2^ where as in another study the mean age of the patients was 48.61±12.36 years old and mean onset was 48.11±12.27 years.^8^ In the same way, in our study the mean ages of the patients was 53.28±14.61 years and 57.81% patient with xanthelasma lesions had duration six months to one year.

Our study found that the mean serum levels of TG 170.29±54.07, TC 200.53±27.09, HDL 42.54 ± 13.94 and LDL 111.31 ± 54.07. The mean serum levels of TG and TC were found to be higher in xanthelasma patients which was consistent with the finding in a study showing higher mean TG (185.98 ±71.7) and TC (221.51 ± 60.4) levels in xanthelasma patients.^2^ A study conducted in Indonesia also reported higher levels of TC (224.61 ±29.77) and TG (167.83±87.26) in xanthelasma patients.^8^

Our study had some limitations. Due to the study design, we could not establish the association of xanthelasma palpebrarum with dyslipidemia. The data was sampled from only one hospital in a defined period of time and during the first visit of the patients. The sample size is limited so it may have some limitations in generalization of results.

## CONCLUSIONS

This study showed that the prevalence of dyslipidemia in patients with xanthelasma palpebrarum was found to be higher than the similar studies done in similar settings. Xanthelasma can act as a marker for dyslipidemia and it might predict the possibility of acquiring coronary artery diseases due to dyslipidemia. Therefore, the occurrence of xanthelasma in an individual should make health workers aware that dyslipidemia and atherosclerosis may be coexisting.

## References

[1] James WD, Berger TG, Elston DM, Odom RB (2006). Andrews' diseases of the skin: clinical dermatology..

[2] Kavoussi H, Ebrahimi A, Rezaei M, Ramezani M, Najafi B, Kavoussi R (2016). Serum lipid profile and clinical characteristics of patients with xanthelasma palpebrarum.. An Bras Dermatol..

[3] Bergman R, Kasif Y, Aviram M, Maor I, Ullman Y, Gdal-On M (1996). Normolipidemic xanthelasma palpebrarum: lipid composition, cholesterol metabolism in monocyte-derived macrophages, and plasma lipid peroxidation.. Acta Derm Venereol..

[4] Jain A, Goyal P, Nigam PK, Gurbaksh H, Sharma RC (2007). Xanthelasma palpebrarum-clinical and biochemical profile in a tertiary care hospital of Delhi.. Indian J Clin Biochem..

[5] Pandhi D, Gupta P, Singal A, Tondon A, Sharma S, Madhu SV (2012). Xanthelasma palpebrarum: a marker of premature atherosclerosis (risk of atherosclerosis in xanthelasma).. Postgrad Med J..

[6] Rudolph RL (1975). Diffuse "essential" normolipemic xanthomatosis.. Int J Dermatol..

[7] Ozdol S, Sahin S, Tokgozoglu L (2008). Xanthelasma palpebrarum and its relation to atherosclerotic risk factors and lipoprotein (a).. Int J Dermatol..

[8] Kampar P, Anum Q, Lestari S (2020). The correlation between lipid profile and xanthelasma.. Berkala Ilmu Kesehatan Kulit dan Kelamin-Periodical of Dermatology and Venereology..

[9] Aggarwal R, Rathore PK (2016). A study evaluating xanthelasma palpebrarum clinically and biochemically.. International Journal of Contemporary Medical Research..

[10] Zhimin W, Hui W, Fengtao J, Wenjuan S, Yongrong L (2020). Clinical and serum lipid profiles and LDLR genetic analysis of xanthelasma palpebrarum with nonfamilial hypercholesterolemia.. J Cosmet Dermatol..

[11] Nair PA, Singhal R (2017). Xanthelasma palpebrarum - a brief review.. Clin Cosmet Investig Dermatol..

[12] Gangopadadhya DN, Dey SK, Chanda M, Pal D, Chaudhuri S (1998). Serum lipid profile in xanthelasma.. Indian Journal of Dermatology..

